# In-roads to the spread of antibiotic resistance: regional patterns of microbial transmission in northern coastal Ecuador

**DOI:** 10.1098/rsif.2011.0499

**Published:** 2011-09-28

**Authors:** Joseph N. S. Eisenberg, Jason Goldstick, William Cevallos, Gabriel Trueba, Karen Levy, James Scott, Bethany Percha, Rosana Segovia, Karina Ponce, Alan Hubbard, Carl Marrs, Betsy Foxman, David L. Smith, James Trostle

**Affiliations:** 1 School of Public Health, University of Michigan, Ann Arbor, MI, USA; 2 Department of Microbiology, Universidad San Francisco de Quito, Quito, Ecuador; 3 Rollins School of Public Health, Emory University, Atlanta, GA, USA; 4 Department of Mathematics and Statistics, Colby College, Waterville, ME, USA; 5 School of Public Health, University of California, Berkeley, CA, USA; 6 Department of Biology and Emerging Pathogens Institute, University of Florida, Gainesville, FL, USA; 7 Department of Anthropology, Trinity College, Hartford, CT, USA

**Keywords:** antibiotic resistance, *Escherichia coli*, transmission models, Ecuador, community

## Abstract

The evolution of antibiotic resistance (AR) increases treatment cost and probability of failure, threatening human health worldwide. The relative importance of individual antibiotic use, environmental transmission and rates of introduction of resistant bacteria in explaining community AR patterns is poorly understood. Evaluating their relative importance requires studying a region where they vary. The construction of a new road in a previously roadless area of northern coastal Ecuador provides a valuable natural experiment to study how changes in the social and natural environment affect the epidemiology of resistant *Escherichia coli*. We conducted seven bi-annual 15 day surveys of AR between 2003 and 2008 in 21 villages. Resistance to both ampicillin and sulphamethoxazole was the most frequently observed profile, based on antibiogram tests of seven antibiotics from 2210 samples. The prevalence of enteric bacteria with this resistance pair in the less remote communities was 80 per cent higher than in more remote communities (OR = 1.8 [1.3, 2.3]). This pattern could not be explained with data on individual antibiotic use. We used a transmission model to help explain this observed discrepancy. The model analysis suggests that both transmission and the rate of introduction of resistant bacteria into communities may contribute to the observed regional scale AR patterns, and that village-level antibiotic use rate determines which of these two factors predominate. While usually conceived as a main effect on individual risk, antibiotic use rate is revealed in this analysis as an effect modifier with regard to community-level risk of resistance.

## 1. Introduction

Antibiotic resistance (AR) threatens human health worldwide [1]. As resistant bacteria spread, and failure of antibiotics in the clinical setting increases in frequency, infections require more expensive drugs and are more likely to be associated with serious morbidity and/or mortality [2]. The cost of these failures exceeds billions of dollars annually in the United States [3]. That the evolution of AR is influenced by individual antibiotic use in human and veterinary medicine is well known [4,5], and programmes aimed at limiting the spread of resistant bacteria often focus on restricting antibiotic use and/or choosing therapeutic options that minimize selection for resistance [6]. Yet, resistance mechanisms are often complex, suggesting that resistant bacteria are not likely to arise by antibiotic selection pressure over the course of treatment alone, and in many cases, the genes that confer resistance must have been acquired by colonizing bacteria or shared among bacteria on mobile genetic elements [7].

The emphasis on evolution of AR during treatment ignores the role of acquisition of resistant bacteria via other transmission routes, such as environmental pathways and human contact patterns. The relative role of these different factors in determining the prevalence of AR within and across communities has not been studied, however, and in general, little is known about the spread of resistant bacteria in community settings. The relationship between the total antibiotic use and the rate of AR spread among individuals in a population is an important, but unresolved question, as is the role of broader ecological processes in spreading resistant bacteria among animals and humans [8,9]. Studying population-level processes shifts the emphasis from individual use to overall antibiotic use rates and the number of other people who carry resistant bacteria [10]. Transmission models are important tools to study such system-level population processes.

Mathematical models of infection transmission have been used throughout the twentieth century to help understand the epidemiology of infectious diseases [11]. These theoretical approaches describe the ecological and evolutionary dynamics of host–pathogen interactions that generate disease patterns in space and time [12]. Mathematical models have been applied to the emergence and the spread of resistant bacteria, extending simple transmission models to reflect competition, such as simple infections with colonization inhibition [13], complex infections with resistance [14] or amplification of resistant bacteria owing to overgrowth following antibiotic use [9]. In general, these models have focused on hospital settings [15] and quantify the effects of different infection control measures [13,16–18]. In hospital settings, healthcare workers are often modelled as vectors that spread resistant organisms among patients [19].

Mathematical models can also offer important insights into the mechanisms and extent of the spread of AR in community settings, which are more difficult to study. Recent AR models have focused on movement of patients among hospitals [19], long-term care facilities [20], and the community [14] and the role of antibiotic use in agriculture [9]. Emergence of AR can be modelled as an invasive pathogen [12] into the human population [9,21] using models that incorporate spatial and social processes [22].

**Figure 1.**
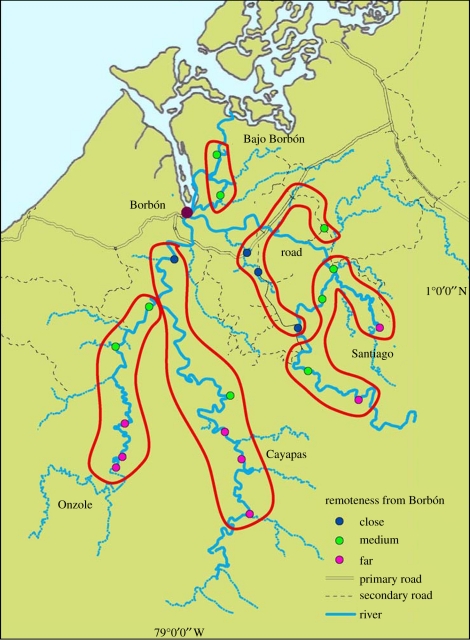
Map of study region. The 21 villages are categorized by river basin (Santiago, Cayapas, Onzole, Bajo Borbón and road), and by remoteness (close, medium and far).

Evaluating the relative importance of individual medication use, environmental transmission and rates of introduction of AR bacteria in explaining community AR patterns requires studying a region where there is variability in all of these factors. The construction of a new road in a previously roadless area of northern coastal Ecuador provides a valuable natural experiment to study how changes in the social and natural environment, mediated by road construction, affect the evolution and the spread of AR enterobacteria. This study area, comprising villages with varying degrees of remoteness relative to the main road (figure 1), offers an ideal location for studying AR at a community scale. Since we postulate that the social and ecological changes that might affect the spread of AR bacteria will unfold over a large time scale, we use remote villages as a proxy for conditions prior to the construction of the road and close villages as a proxy for conditions after. We, therefore, use a cross-sectional design along with statistical models to examine AR as a function of remoteness, and we use mathematical models to explain the relative contributions of: (i) antibiotic use; (ii) transmission of AR bacteria, generally mediated through standard water, sanitation and hygiene environmental pathways; and (iii) rates of introduction of resistant bacteria, represented in our model as an ingestion factor, in explaining observed patterns of AR in 21 communities. The spread of resistant bacteria is framed here as a spatially inhomogeneous process that affects prevalence. This occurs through both environmental sources and human movement patterns, whose effects are modified by conditions that increase the potential for human-to-human transmission, such as poor sanitation. Based on 5 years of data across 21 communities, we describe regional patterns of AR prevalence and use a transmission model to provide plausible explanations for these observed patterns.

## 2. Methods

### 2.1. Study site

In the northern Ecuadorian province of Esmeraldas, approximately 150 villages (ranging from 20–800 inhabitants) lie along the Cayapas, Santiago and Onzole rivers, which all flow towards Borbón, the main population centre of the region (with 5000 inhabitants). Villagers primarily consume untreated surface source water and sanitation facilities are inadequate. The region, populated primarily by Afro-Ecuadorians [23], is undergoing intense environmental and social changes owing to the construction of a new highway along the coast, which connects previously remote villages to the outside world. Construction of the road was completed from Borbón westward to the provincial capital of Esmeraldas in 1996 and from the coast eastward to the Andean mountains in 2003. Secondary and tertiary dirt roads off of this two-lane asphalt highway are continually being built, mostly for logging and the area has come to be known as one of the world's top 10 biodiversity hotspots [24]. At the time these data were collected, 15 per cent of the 150 villages in the region were accessible by road.

All villages in the region were categorized based on their geographical location relative to Borbón. A sample of 21 villages was selected by using block randomization to ensure that villages of varying remoteness and population sizes were represented; four of these were connected to the road when this study began. All households within each village were recruited, except in Borbón, where a random sample of 200 households (from approx. 1000) was selected for inclusion in the study. Consent was obtained at both the village and household level. Institutional review boards at the University of California Berkeley, University of Michigan, Trinity College and Universidad San Francisco de Quito approved all protocols.

### 2.2. Study design

Between August 2003 and February 2008, each enrolled village was visited seven times, with each visit lasting 15 days. Villages were visited on a rotating basis, during which time field staff identified all cases of diarrhoea through active surveillance. For each case of diarrhoea (defined using WHO standards as three or more loose stools in a 24 h period), two controls were randomly sampled from the same community, and one control was sampled from the case household. Controls were defined as someone with no signs of diarrhoea in the previous 6 days. Four 15 day case–control visits were conducted in Borbón. Antibiotic usage was measured through a sequential random sample of households where many of the households were measured more than once. A key informant was asked whether any household members had used antibiotics within the last week and, if so, they were asked to name the drug. Responses from the key informant were converted to the individual level by recording usage for those identified by the survey and imputing a response of ‘No usage’ for the remaining individuals who were known to live in the house from previous demographic surveys.

### 2.3. Classifying remoteness

For each village, travel time and total cost of travel to Borbón were recorded by field staff members. Specifically, transport time was estimated assuming the use of a motorized canoe or bus, depending on location, and transport cost was determined through inquiries of key informants within each community. For each village, *i*, rank of remoteness, *R*_*i*_, was calculated by summing normalized values of time, *T*_*i*_ and cost, *C*_*i*_. Specifically,
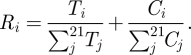


Since the metric is the result of two values standardized to a [0,1] scale, the possible range of *R*_*i*_ is from 0 (the town Borbón itself) to 2 (the theoretical farthest community from Borbón). Villages were classified into three groups based on their remoteness metric: close, medium and far from Borbón. The categorical breakpoints were selected by maximizing the differences in the mean remoteness values for each category.

### 2.4. Microbiological analysis

Stool samples were collected by field staff from cases and controls, stored on ice and processed within 48 h and tested for the presence of *Escherichia coli* and AR. Lactose positive isolates that were identified as *E. coli* were further analysed for antibiotic susceptibility (to ampicillin (amp), cefotaxime, chloramphenicol, ciprofloxacin, gentamicin, sulphamethoxazole–trimethoprim (sxt) and tetracycline) using the disc-diffusion method following standard methods. To test for the presence of *E. coli*, stool was plated directly onto MacConkey agar; lactose positive colonies were further cultured in Chromocult agar. The five most prominent lac+ isolates were initially selected and one confirmed *E. coli* isolate was randomly chosen for further AR analysis. All lactose negative isolates were analysed for urease and oxidase, and with API 20 E (bioMérieux Corp) to speciate the bacterial isolates. Lactose positive isolates that were identified as *E. coli* were further analysed for antibiotic susceptibility (to amp, cefotaxime, chloramphenicol, ciprofloxacin, gentamicin, sxt and tetracycline) using the disc-diffusion method following standard methods [25,26]. As sulphamethoxazole and trimethoprim work synergistically, they are commonly used together, often in the same pill. Therefore, one standard clinical approach is to screen for the combined resistance to both at the same time with discs impregnated with both antibiotics, and the resulting resistance to both antibiotics is then listed as sxt resistance. This was done as part of this study, with the limitation that we do not have information on *E. coli* isolates that were resistant to sulphamethoxazole, but not trimethoprim, or vice versa. These seven antibiotics were chosen to be included in this study because they were reported to be the most commonly used antibiotics in the region both by physicians within our field staff and by other physicians who also work in the study region.

### 2.5. Statistical analysis

Our statistical analysis consists of the following: (i) calculating prevalence of each AR profile correcting for the unequal sampling probabilities of cases and controls; (ii) estimating the variability of individual-level antibiotic use using random effects models to compare variability over time over space; (iii) estimating the association between AR and remoteness using binary response general estimating equation (GEE) models; (iv) summarizing prevalence of antibiotic use in terms of drugs most frequently used, and in terms of prevalence of use; (v) exploring how antibiotic use rates vary as a function of remoteness to investigate their potential utility in explaining observed AR patterns; and (vi) examining the assumptions associated with aggregating our AR data over time.

#### 2.5.1. Calculating prevalence

The data used to estimate the distribution of AR was a non-standard case–control design consisting of cases, household controls and community controls. Since cases are relatively rare, simple estimators of prevalence are potentially biased owing to over-representation of cases. To obtain community prevalence estimates, therefore, required different analytical techniques that use the following weighting procedure. Cases (those presenting with diarrhoea) were given a weight of 1, since all cases in each community were sampled, giving them a sampling probability of 1. Household controls (those sampled within a house with a case and not presenting with diarrhoea) are weighted by the inverse of the proportion of the susceptible population of household controls represented by the control sample. The equivalent weight is also calculated for the community controls (note, this weighting was done by community and collection cycle, and thus the weighted contribution of a community/cycle to the analysis is the same regardless of its total population size, i.e. the communities are the units). Using these weights, we calculate the standard Horvitz–Thompson estimator [27] of prevalence, which yields unbiased estimators of population means and proportions in unequal probability samples.

#### 2.5.2. Variability of antibiotic use

To compare the variability of antibiotic use over time and over space, two random effect models are fit with antibiotic use as the dependent variable. In the first model, the variance of the random offset corresponding to household is estimated; in the second, the variance of the random offset corresponding to time point. Comparison of the size of these variances is then used to give an indication of whether there is more variability between households (spatial) or between time points (temporal). Further details on this analysis are given in the electronic supplementary material.

#### 2.5.3. Association between antibiotic resistance prevalence and remoteness

To explore the relationship between amp–sxt resistance prevalence and remoteness, we estimate the odds ratio between the binary indicator of amp–sxt resistance and (i) the binary indicator of medium/close remoteness, using ‘far’ as the reference category as well as (ii) the binary indicator of residence in Borbón using the other communities as the reference category. To correct for unequal probability sampling, each observation is replicated a number of times equal to its sampling weight. Odds ratios are estimated by fitting a logistic regression model to this expanded dataset. To derive the statistical inference for the relevant measures of association, we relied on the clustered non-parametric bootstrap, specifically re-sampling 21 villages with replacement from the expanded dataset and estimate the odds ratio from this ‘bootstrap dataset’ [28]. This process is repeated 10 000 times to estimate the sampling distribution of the odds ratios and we use the quantile method to derive the 95% CI. In the far versus medium/close comparisons, only bootstrap datasets that have at least two villages from each remoteness category are included, since the sampling of villages was done to create variability between villages in terms of remoteness. Therefore, datasets with 1 or 0 villages in one or more remoteness categories do not reflect the sampling distribution of interest. Similarly, in the Borbón versus community comparisons, bootstrap datasets that did not include Borbón at least once did not contribute to the reported confidence intervals (CI).

#### 2.5.4. Prevalence of antibiotic use

To characterize the per-day prevalence of use, we calculate the proportion of individuals that report having used antibiotics within the last week and scale this quantity by 7, tacitly assuming that individuals only used drugs on 1 day within the last week and it was equally likely to have been any day. Since individuals could have ingested drugs on more than 1 day, our use rate constitutes a lower bound. To look at what drugs are most commonly used, we summarize the relative frequency of drugs used among those that reported use (electronic supplementary material, table S1).

#### 2.5.5. Antibiotic use and remoteness

Antibiotic use at a community level is estimated by the sample proportion of respondents who reported using antibiotics. We consider an individual to have used antibiotics if they indicate they have consumed any of: amp, amoxicillin, sulphamethoxazole, trimethiprim or benzipenicillin. Ordinary least-squares regression was used to look at the relationship between the village-level proportion and remoteness. Although the proportion reporting use is clearly bounded between 0 and 1, the discrepancies from the regression line appeared symmetric (electronic supplementary material, figure S2), making ordinary least squares a tenable choice.

#### 2.5.6. Aggregation of antibiotic resistance data over time

To justify that the effect of remoteness on amp–sxt resistance is static, this relationship is assessed at each of the seven time points using a GEE model in the same way as was done in estimating the relationship between remoteness and AR prevalence on the full dataset. For each time point separately, we fit an independence GEE model with remoteness category as the lone predictor and amp–sxt resistance as the response variable. Confidence intervals for the odds ratios comparing ‘far’ with the two other categories were produced using the same non-parametric bootstrap described for the full dataset, and intervals were examined for overlap (electronic supplementary material, figure S1). Greater detail is given in the electronic supplementary material.

### 2.6. Modelling

**Figure 2.**
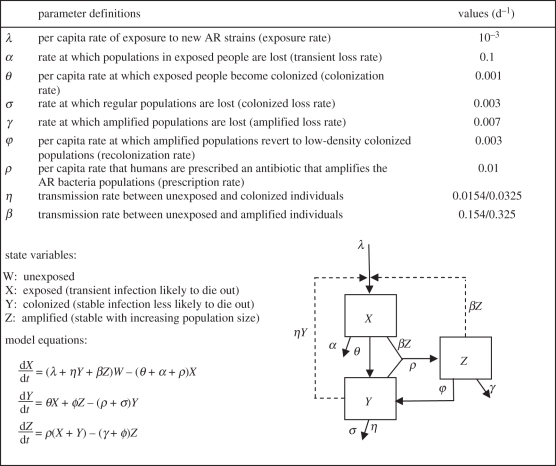
Deterministic antibiotic resistance model. *W* = 1−*X*−*Y*−*Z*. See Smith *et al*. [9] for details.

We use a village-level compartmental transmission model [9] to examine the observed patterns of AR prevalence in our study communities. We chose a compartmental model, which assumes populations are well-mixed, because it provides better explanatory power than more complex model structures for understanding factors that drive transmission. A deterministic model does not allow for the possibility of stochastic die-out, but at the phenotypic level, we do not observe this; i.e. all communities have non-zero prevalence. At the genotypic level, there could be stochastic die-out of specific strains, however, we do not have the genotypic information to illustrate this and therefore did not include this level of resolution in the model. The equation and parameters are shown in figure 2. This model tracks four conditions among humans: (i) not colonized with resistant bacteria (*W*); (ii) transiently colonized with resistant bacteria, such that the bacteria have a high probability of dying out (*X*); (iii) colonized with resistant bacteria such that the population is more stable and less likely to die out compared with the exposed state (*Y*); and (iv) amplified or colonized with resistant bacteria such that bacterial species are present in high numbers and are actively reproducing (*Z*).

The model assumes that human exposure to resistant bacteria comes from either: (i) the spread of these AR bacteria through standard water, sanitation and hygiene pathways, or (ii) the ingestion of new antibiotic resistant strains that arise from either environmental sources (e.g. food or water) or introduction through movement of people to and from the region. AR spread is modelled as person-to-person transmission. Amplified resistant bacteria (*Z*) are assumed to transmit at a higher rate, **β**, than the unamplified or colonized bacteria, which transmit at a rate **η**. The rate of ingestion is described by the parameter **λ**. Antibiotic use, at a rate **ρ**, is assumed to alter the community ecology of the gut, eliminating competition with antibiotic-sensitive bacteria and allowing the population density of resistant bacteria to increase. The remaining five parameters that represent the rates of movements between states are described in figure 2 as well as in Smith *et al*. [9].

Although we observe that cases have higher prevalence of AR than do controls, both cases and controls have higher prevalence of AR in the less remote villages. Thus, in the simulation analysis, we do not make a distinction between cases and controls.

An estimate of the transmission rate was established using *E. coli* prevalence data from our study region. Previous analysis of these data suggests an eightfold difference in *E. coli* prevalence comparing remote versus non-remote villages [29]. We use the prevalence values from this analysis for these two types of villages in conjunction with a susceptible–infected–susceptible (SIS) model (with disease duration of one week) to estimate **β**, the rate of transmission from amplified to susceptible individuals. **β** is estimated to be 0.154 new infections per infectious individual per susceptible individual per day for the most remote village, and 0.325 for the least remote village, a transmission rate ratio of 2.11. To explore the sensitivity of transmission to AR prevalence, we vary this ratio in our simulation analysis from 0.9 to 9 keeping the baseline transmission rate for remote villages at 0.154. The rate of transmission from colonized to susceptible individuals, **η**, is assumed to be one-tenth the value of **β** because colonized individuals have smaller populations of AR bacteria in their gut than amplified individuals.

The antibiotic use rate, **ρ**, is based on survey data collected in each village, and does not vary by remoteness. The antibiotic use data, employed to estimate the antibiotic use rate parameter, **ρ**, are presented in §3. We specify the range for the antibiotic use rate by extending the 95% CI, resulting in the range **ρ**: 0.001 to 0.01 antibiotics per person per day.

The *per capita* rate of human exposure to new strains (introduction rates), **λ**, is unknown for this region. We use the same per day baseline rate (0.001) reported in Smith *et al*. [9] to represent a remote village. To explore the sensitivity of **λ** to AR prevalence, we vary the rate of non-remote villages so that the ratio ranges from 1 to 10. The assumption that introduction rates are higher in non-remote villages is consistent with the observation that there is more human movement to and from outside the region in these non-remote villages [29], providing more opportunity to introduce AR bacteria.

To examine the interaction between antibiotic use rates, transmission rate ratios comparing remote and non-remote villages, and introduction rate ratios comparing remote and non-remote villages, we simulate the model for a range of each of these three factors and use contour plots to present their relationship. The outcome measure is the risk ratio comparing a remote to a non-remote village. This risk ratio measure was compared with the empirical results presented in table 2.

## 3. Results

Between 2003 and 2008, a total of 2210 *E. coli* isolates were successfully analysed (518 were cases with diarrhoea and 1692 were controls without diarrhoea). We stratify our analysis by case/control status since the microbiota of those with diarrhoea is quite different from those without diarrhoea. Using results of screening isolates for sensitivities to seven antibiotics, we observed 39 unique profiles. The nine highest frequency profiles are listed in table 1. The distribution of antibiotic profiles differs between cases and controls with cases having a tendency towards a higher frequency of resistance. Three of the most frequently observed profiles include resistance to amp and sxt. Sulphamethoxazole-resistant genes and trimethoprim-resistant genes are almost always present on the same integrons, while β-lactamase genes encoding resistance to amp can sometimes also be found in the same integron [30,31] or outside of the integron, but on the same plasmid [32,33]. In contrast, tetracycline resistance is never found as part of an integron [34]. Thus, amp and sxt resistance are more likely to be horizontally and clonally transmitted together. For this reason, and because antibiotics that select for amp–sxt resistance are frequently used in the region (see below), we focus analysis on amp–sxt.

**Table 1.** Estimated prevalence, weighted by the inverse sampling probability, of antibiotic-resistant *E. coli* profiles. Cases are defined as those with diarrhoea and controls are those without. All profiles with frequencies of less than 1% are placed in the ‘other’ category. The antibiotics tested are: ampicillin (amp), tetracycline (tet), sulphamethoxazole–trimethoprim (sxt), chloramphenicol (clo), cefotaxime (ctx), gentamicin (gen) and ciprofloxacin (cip).

profile	prevalence (per 100)		
	total	cases	controls
none	67.5	51.5	67.8
amp–sxt–tet	8.0	19.8	7.8
tet	6.9	4.0	7.0
other	3.5	4.7	3.5
amp	3.0	3.5	3.0
sxt–tet	2.9	1.8	2.9
amp–sxt–tet–clo	2.6	4.6	2.6
amp–tet	2.3	3.5	2.3
amp–sxt	2.1	6.3	2.1
sxt	1.0	0.3	1.1

We first report on the relationship between remoteness and amp–sxt resistance, showing that amp–sxt resistance decreases with remoteness. We next present our data on antibiotic use and show that there is no relationship between antibiotic use and remoteness, suggesting that the relationship between AR prevalence and remoteness cannot be explained by differences in use rates alone. We present the results of an infection transmission model that examines the interaction between antibiotic use, transmission of resistant bacteria and introduction of resistant bacteria into villages in determining regional patterns of AR. The model analysis suggests that patterns of transmission as well as patterns of introductions of resistant bacteria into communities contribute to the regional-scale AR patterns we observed, and that antibiotic use rates determine which of these two factors predominate.

### 3.1. Ampicillin–sulphamethoxazole–trimethoprim resistance as a function of remoteness

Ampicillin–sulphamethoxazole–trimethoprim (amp–sxt) resistance is significantly associated with lack of remoteness (table 2). This trend is consistent for both cases and controls. Estimating the community prevalence based on a weighted sum of the case and control observations, there was little difference in villages of far and medium remoteness (OR = 1.1 [0.6, 1.8]) whereas close villages have higher prevalence relative to far villages (OR = 1.8 [1.3, 2.3]). Similarly, there are higher levels of resistance in Borbón, the main population centre of the region, compared with the communities collectively OR = 1.3 [1.1, 1.6]). Although data were observed at seven different time points, we aggregate the data in this analysis, making an assumption about temporal stability of these relationships. This assumption is supported by data presented in electronic supplementary material, figure S1, which show that the confidence intervals for the odds ratios stratified by time overlap.

As with any symptom-based definition, there is the possibility of misclassification; however, if we assume that the disease misclassification is non-differential across our exposure (in this case remoteness of our study villages), then misclassification will bias the results towards the null. We would, therefore, expect greater differences among our remoteness categories if we could adjust for this bias.

**Table 2.** Prevalence and odds ratio of simultaneous antibiotic resistance to amp and sxt among participants living in 21 villages in Ecuador. Cases are defined as those with diarrhoea and controls are those without. Medium and close categories are compared with the far category. Observations are weighted based on their inverse sampling probability to account for unequal probability sampling.

remoteness	sulphamethoxazole and ampicillin resistance			
	case prevalence (infections per 100)	control prevalence (infections per 100)	overall prevalence (infections per 100)	OR (95% CI)
far	35.2	12.4	12.8	1.0
medium	32.6	13.4	13.8	1.1 (0.6, 1.8)
close	43.0	20.1	20.5	1.8 (1.3, 2.3)
community	37.6	15.6	16.0	1.0
Borbón	46.4	19.4	20.0	1.3 (1.1. 1.6)

### 3.2. Antibiotic use

During the study period, we surveyed 1875 individuals about their antibiotic use in a population that averaged around 4000 at any given time. On average, each sampled individual was surveyed 1.3 times over the study period, ranging from one to six times, also resulting in multiple measurements of each household. A random effects analysis of these data supports our sampling strategy for added coverage across households rather than coverage over time (see electronic supplementary material). Among those individuals reporting use, the most frequently named antibiotics were amoxicillin (20% of antibiotics mentioned), amp (13%), sulphamethoxazole/trimethoprim (8%) and ciprofloxacin (8%) (electronic supplementary material, table S1). In the analysis presented in this manuscript, we restrict focus only to drugs that select for amp–sxt resistance. In addition to its constituent drugs, we also include amoxicillin and benzylpenicillin. These are in the family of beta lactams and therefore their use potentially selects for amp resistance. Over the 5 years of collecting survey data across the region, the average use rate was 0.05 per individual per week. Assuming use is evenly distributed throughout the week, this corresponds to a rate of 0.006 per individual per day, with an associated 95% CI of (0.003, 0.010). This rate per day is used in our subsequent simulation studies. There was no relationship between antibiotic use and remoteness at the community level (see electronic supplementary material, figure S2).

### 3.3. A transmission perspective on the observed antibiotic resistance patterns

We use a transmission model to examine how the interaction among antibiotic use, transmission rates of antibiotic resistant *E. coli* and introduction rates of antibiotic resistant *E. coli* into villages affect the community-level AR patterns that we observed. As described in the transmission model, the transmission and introduction rates vary by remoteness, whereas antibiotic use does not. Our transmission model analysis suggests that the level of antibiotic use determines which factors explain the risk ratio of AR prevalence when comparing a close village with a far village: the ratio of transmission rates (close versus far) and/or the ratio of introduction rates (close versus far). This result is shown using contour plots of the risk ratio as a function of both the transmission rate and introduction rate ratios for both low and high antibiotic use rates (see electronic supplementary material, figure S3).

**Figure 3.**
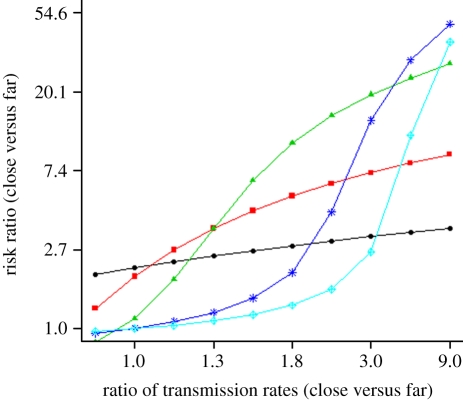
The risk ratio of AR prevalence comparing a non-remote village (close) with a remote village (far) as a function of the ratio of transmission rates for close versus far villages. Each plot is for a different antibiotic use rate (**ρ**) ranging from 0.001 to 0.01 antibiotics per person per day. The transmission rate of the remote village is 0.154 (see text for justification). See figure 2 for remaining parameter values. Circles with solid line, **ρ** = 0.001; squares with solid line, **ρ** = 0.002; triangles with solid line, **ρ** = 0.003; asterisks with solid line, **ρ** = 0.006; diamonds with solid line, **ρ** = 0.01.

To examine the marginal effects of transmission ratio and antibiotic use rate, we integrate out the introduction rate by calculating the geometric mean of the observed risk ratios across all introduction rate values (figure 3). This is virtually identical to risk ratios corresponding to fixing the introduction rate ratio to its midpoint value of two. Figure 3, therefore, presents a plot of the effect of the ratio of transmission rates in close versus far communities on the risk ratio for sxt–amp resistance in close versus far communities for various antibiotic use rates to display the interaction between use rate and transmission ratio (figure 3). For extremely low antibiotic use rates (e.g. **ρ** = 0.001 per day), the transmission rate ratio has little effect on the risk ratio; i.e. given little selection pressure on AR in the village, transmission cannot amplify the prevalence levels. Under this scenario, the prevalence differences among villages can be attributable to differences in the introduction rates of resistant bacteria. The transition from no relationship to a very strong relationship between the transmission ratio and risk ratio can be seen as **ρ** increases. As this happens, the transmission rate ratio becomes the predominant determinant of the risk ratio; i.e. antibiotic use selects for AR and resistant bacteria spread throughout the villages via transmission pathways. It appears that in our study region, AR prevalence is most sensitive to changes in the transmission rate ratio. This conclusion is based on our site-specific estimates of: (i) **ρ** (0.003 to 0.01); (ii) the ratio of the transmission rate, **β**, comparing close versus far villages (2.11); and (iii) the risk ratio of AR prevalence (1.8 [1.3, 2.3]).

## 4. Discussion

Roads have important impacts on social and ecological processes that in turn have impacts on health [35]. The relationship between roads and disease has been examined for a variety of infectious diseases including HIV, malaria, dengue and diarrhoeal disease [29,36–38]. Here, we provide data from a 5 year regional-scale observational study showing that roads can also impact the spread of resistant bacteria. Focusing on *E. coli* resistance to amp–sxt, the most common pairing of antibiotics observed, we found a higher prevalence of antibiotic-resistant bacteria in villages along the road compared with more remote villages. These results are consistent with those of other researchers, who have found higher levels of AR organisms in sites with greater anthropogenic influence [39–42].

However, we found no relationship between antibiotic use and remoteness, which probably relates to the presence of both governmental and non-governmental organizations that deliver medical care, including antibiotics, throughout the region. Given its homogeneous distribution along the remoteness gradient, we employed a village-level transmission model to better understand how antibiotic use impacts prevalence patterns at a regional scale. Our model analysis suggests that at the regional-scale individual antibiotic use serves to modify the effect of two potentially important processes: the transmission of *E. coli* from person to person mediated through environmental pathways, and the introduction of *E. coli* from outside the region owing to the movement patterns of people into and out of the region [29]. As antibiotic use rates decrease across the region, the differential rate of introduction becomes a more important determinant of our observed prevalence patterns. Transmission becomes an important determinant when antibiotic use increases; i.e. antibiotic use amplifies transmission. Thus, antibiotic use has a regional-scale impact that differs from those impacts that are derived from only considering the individual-level scale.

At the individual scale, experimental evidence suggests that resistant bacteria can be out-competed by their sensitive counterparts [43]. The implication of this is that once the pressure of antibiotics is removed, the population of resistant bacteria may decrease relatively quickly, making an individual's antibiotic use act primarily as a main effect on his/her probability of colonization with a resistant strain. However, at the community level, the effect of antibiotic use is more complex. Evidence suggests that the fitness costs of resistance can be very low [44–46], and therefore the subsequent slow decline in the prevalence of resistant bacteria once the antibiotic use ceases, provides continued opportunity for resistant organisms to spread from host to host, from host to the environment and from the environment to the host. Therefore, interplay between antibiotic use, disease transmission rate and rate of introduction from the environment must be considered when characterizing drivers of population-level prevalence of resistant bacteria.

Our analysis suggests that the antibiotic use rate acts to modify the impact of the transmission rate and outside introduction rate, indicating that the effect of antibiotic use rate on community-level prevalence cannot be thought of in isolation. When antibiotic use is high (e.g. *ρ* = 0.01, antibiotics per person per day), the bacteria resistant to the antibiotic being used is selected for within the individual, thereby making it more likely for a transmission event to involve a resistant organism. Under these conditions, transmission becomes a major driver of AR prevalence, with outside introduction having a comparatively very small effect. When antibiotic use is low (e.g. *ρ* = 0.001, antibiotics per person per day), most transmission events involve sensitive bacteria, rendering the transmission rate impotent as a driver of AR prevalence (figure 3). In this setting, oral exposure, which occurs through ingestion of bacteria into the gastrointestinal tract, is the primary driver of prevalence; this exposure comes from a variety of sources including introduction from outside the region. Many studies have demonstrated that AR can spread between individuals sharing the same home [47], day care centre [48,49] or even community [50]. For enteric organisms both transmission and outside introduction occurs through water, sanitation, hygiene and food pathways— modes of spread especially strong in agricultural settings [51] and developing countries [52]. The transmission of bacteria can occur through these pathways in developed countries as well, albeit at lower rates.

Typical models of AR are set in controlled environments such as hospitals, and focus on the competitive advantage given to resistant bacteria through antibiotic use. In such models, invasion of resistant bacteria from the outside is ignored, potentially because the focus of hospital settings is on the large amounts of antibiotic use and how they are optimally prescribed (e.g. [13]). On the other hand, in a community setting, the invasion and the spread of resistant bacteria are an important determinant of prevalence. The inclusion of the rate of introduction of antibiotics and its interaction with transmission and antibiotic use, therefore, is a central piece of our analysis.

The complete understanding of the dynamics of AR spread in the context of social and ecological changes can only be obtained through a systematic and ecological perspective as presented in this study. Our data and analysis support the proposal that understanding the mechanisms of the evolution and the spread of resistant bacteria require a consideration of the ecological dynamics that shape microbial population structure [22]. These dynamics are mediated through factors that determine selection pressures, routes of transmission and the invasion of resistant bacteria [22], which may overwhelm the direct effects of individual antibiotic use in determining the emergence and dissemination of AR across communities or regions. In our study region, the major driver of selection pressure and routes of transmission appears to be a new network of roads, which have strong influence on the social and ecological environment and in turn on the health of communities [37,38,53,54]. Roads may affect the evolution and the spread of resistant bacteria by influencing the use of antibiotics in the human population, changing hygiene and sanitation and introducing resistant bacteria when people travel or migrate into a region.
